# PREDICT-GBM: A multicenter platform advancing personalized glioblastoma radiotherapy planning

**DOI:** 10.1038/s41746-026-03194-0

**Published:** 2026-09-09

**Authors:** Lucas Zimmer, Jonas Weidner, Michal Balcerak, Florian Kofler, Mara Krupa, Ivan Ezhov, Santiago Cepeda, Ray Zirui Zhang, John S. Lowengrub, Bjoern Menze, Benedikt Wiestler

**Affiliations:** 1 AI for Image-Guided Diagnosis and Therapy, Technical University of Munich, Munich, Germany; 2 Munich Center for Machine Learning (MCML), Munich, Germany; 3 Department of Quantitative Biomedicine, University of Zurich, Zurich, Switzerland; 4 Helmholtz AI, Helmholtz Zentrum München, Munich, Germany; 5 Hertie Institute for AI in Brain Health, University of Tübingen, Tübingen, Germany; 6 AI in Healthcare and Medicine, Technical University of Munich, Munich, Germany; 7 Department of Neurosurgery, Río Hortega University Hospital, Valladolid, Spain; 8 Specialized Group in Biomedical Imaging and Computational Analysis (GEIBAC), Instituto de Investigación Biosanitaria de Valladolid (IBioVALL), Valladolid, Spain; 9 Department of Mathematical Sciences, Worcester Polytechnic Institute, Worcester, MA USA; 10 Department of Mathematics, University of California, Irvine, CA USA; 11 Department of Biomedical Engineering, University of California, Irvine, CA USA

**Keywords:** Cancer, Computational biology and bioinformatics, Oncology

## Abstract

Glioblastoma recurrence is largely driven by diffuse infiltration beyond radiologically visible margins, yet current radiotherapy guidelines rely on uniform margin expansions that ignore patient-specific biology and anatomy. While computational models promise to map this invisible growth and guide personalized planning, their clinical translation is hindered by a lack of standardized benchmarking and reproducible validation. To bridge this gap, we present PREDICT-GBM, an open-source platform integrating a curated, longitudinal, multi-center dataset of 243 patients with a standardized evaluation pipeline. We benchmark a novel U-Net-based recurrence prediction model against state-of-the-art biophysical and data-driven methods. Under iso-volumetric constraints, both biophysical and deep-learning approaches achieved modest but statistically significant gains in geometric coverage of future recurrence over guideline-based plans. On the combined cohort, our U-Net achieved the highest mean coverage of enhancing recurrence (79.37 ± 2.08%), surpassing guideline-based plans (paired Wilcoxon signed-rank test, Benjamini-Hochberg adjusted *p* = 2.9 × 10^−5^). The biophysical model GliODIL reached 78.91 ± 2.08% (*p* = 1.0 × 10^−3^), validating the platform’s ability to compare diverse modeling paradigms. By providing a reproducible ecosystem for model training and validation, PREDICT-GBM addresses a major bottleneck toward personalized, computationally guided radiotherapy. The platform, models, and data are openly available at github.com/BrainLesion/PredictGBM.

## Introduction

Glioblastoma (GBM) remains one of the greatest challenges in oncology, defying modern therapeutic advances, with median overall survival (OS) stagnating at 14–16 months since the introduction of adjuvant temozolomide to standard post-operative radiotherapy (RT) in 2005^1–6^. Glioblastoma is characterized by its highly invasive nature, with tumor cells diffusely infiltrating the surrounding brain parenchyma, leading to near-universal tumor recurrence despite aggressive, multimodal therapy^7,8^. Standard-of-care treatment consists of maximal safe resection followed by adjuvant RT with concomitant and adjuvant chemotherapy^9^. MRI is central to both treatment planning and longitudinal disease monitoring. Because MRI only delineates regions of high tumor cell density, and even advanced MRI struggles to delineate the diffuse infiltration of tumor cells into the surrounding parenchyma^10^, microscopic tumor infiltration is addressed in clinical practice by applying a uniform margin to the visible tumor to define the clinical target volume (CTV) for irradiation, in accordance with current RT guidelines^8^. However, this margin-based approach does not capture patient-specific tumor biology, invasion patterns, or anatomy. Consequently, this “one-size-fits-all” approach risks simultaneously undertreating the tumor and overtreating the surrounding brain.

To enable the true personalization of RT volumes, computational models of glioblastoma have been proposed. These models simulate tumor growth and thereby generate three-dimensional tumor cell density distributions beyond the margins visible in standard MRI, with the potential to support biologically informed, patient-specific RT target definition. Early approaches predominantly relied on reaction-diffusion models of tumor cell proliferation and migration, solved using PDE-constrained optimization frameworks incorporating Bayesian inference^11–14^, numerical solvers^15–19^, or analytical approaches^20^. High computational cost and limited availability of suitable longitudinal imaging data have historically restricted these studies to small patient cohorts. Subsequent work leveraged the advances of deep learning for data-driven approaches either by learning the forward problem to optimize a surrogate model^21–23^ or by learning the inverse problem, mapping from images to growth parameters in the PDE^24–26^. Recent work further combines model-driven and data-driven principles by learning a prior for the growth parameters before utilizing evolutionary sampling with a numerical solver^27^, introducing a neural PDE solver by performing gradient-based optimization with respect to the growth parameters during inference^28^, or introducing a physics term in the loss function^29–33^. Another recent line of work frames the problem as direct recurrence prediction, bypassing explicit estimation of a tumor cell density map and instead relying on purely data-driven learning approaches^34–36^.

Despite these promising methodological advances, the clinical translation of glioblastoma growth models remains limited. Successful translation requires comparative validation across sufficiently large and heterogeneous patient cohorts; however, prior research has predominantly relied on small, institution-specific datasets without standardized evaluation protocols. Only a limited number of studies report evaluations on cohorts exceeding 100 patients^17,19,30,32^. This fragmentation stems largely from practical barriers to data access. Robust assessment of growth models necessitates longitudinal imaging, specifically, pre-operative MRI and follow-up scans capturing the first tumor recurrence, which poses significant curation challenges. Furthermore, sharing such high-resolution cranial MRI data is often restricted by privacy and anonymization concerns. While platforms like The Cancer Imaging Archive (TCIA)^37^ provide valuable access to de-identified glioblastoma datasets, few publicly available collections meet the specific longitudinal criteria required for growth modeling. Consequently, the field lacks a standardized benchmark, rendering meaningful comparison between distinct modeling approaches impossible.

In addition to data scarcity, the lack of a standardized processing pipeline introduces significant variability. Most models require co-registered, multi-sequence MRI inputs, along with segmentations of both tumor and healthy brain tissue. While the multimodal Brain Tumor Segmentation (BraTS) benchmark has established standards for tumor segmentation^38–42^, extracting the healthy tissue geometry required to model cell migration remains an open challenge. General-purpose neuroimaging tools, such as FreeSurfer and SynthSeg^43,44^, FastSurfer^45^, FSL FAST^46^, or ANTs^47^, offer solutions for healthy brains but often struggle in the presence of significant pathology. Specifically, accurate tissue classification in the peritumoral region is confounded by mass effect, edema, and post-operative changes. As a result, researchers often rely on custom, unstandardized preprocessing workflows. Lastly, there is no consensus on the evaluation metrics. This leads to slightly different definitions of, e.g., “recurrence coverage” and related concepts across studies, effectively precluding the community from objectively comparing different approaches to modeling and predicting tumor growth for personalized therapy. These disparities in data curation, image processing, and evaluation metrics highlight the critical need for an open, extensible platform to unify and accelerate model development.

To address these challenges, we introduce PREDICT-GBM: the first open, extensible platform for glioblastoma growth models and recurrence prediction models, designed to enable standardized evaluation and facilitate their translation to clinical application. PREDICT-GBM provides a large, curated, and fully processed longitudinal glioblastoma imaging dataset, together with a unified evaluation pipeline that enables head-to-head comparison of state-of-the-art computational models against each other and against a guideline-based comparator plan, and provides a critical resource for the development and evaluation of new approaches to personalized glioblastoma treatment. In the following, we refer to the guideline-based comparator plan as SOC, but emphasize that this represents a simplified, uniform-margin expansion evaluated geometrically on pre-operative anatomy in contrast to dose-aware standard-of-care planning on post-operative anatomy. By effectively comparing diverse, modeling- and learning-based approaches, PREDICT-GBM provides critical insights into the state of the field and future research directions. The entire framework is fully open source, allowing researchers to process additional datasets, develop and benchmark new growth models, and perform reproducible evaluations in a simple, standardized manner.

## Results

### Patient characteristics

The combined PREDICT-GBM cohort included 243 subjects, curated from three datasets (LUMIERE^48^, RHUH^49^, and TUM-GBM) that met our data inclusion criteria. Patient characteristics are summarized in Table 1. The median baseline tumor volume was 27.5 cm^3^, while the median recurrence volume was 5.6 cm^3^, with cases spanning a broad range of lesion sizes across the cohort, well in line with the broad heterogeneity seen in clinical routine. To contextualize spatial patterns, Table 1 also reports the distance between the primary tumor and the recurrence, measured between their centers of mass (CoM). Recurrences were predominantly local, a well-known finding in glioblastoma^50^, with a median center of mass (CoM) distance of 1.6 cm, while the cohort also included more distant recurrences with CoM displacements of up to 9.1 cm.

**Table 1:** Patient characteristics

Patient cohort	All patients	RHUH	LUMIERE	TUM-GBM
***N***	243	40	61	142
**Tumor volume** (**cm**^3^)				
Average/stdev	32.3 ± 26.2	35.9 ± 27.1	33.8 ± 25.0	30.7 ± 26.4
Median	27.5	31.4	27.5	25.8
IQR	[12.2 - 47.3]	[12.3–52.9]	[13.9–49.8]	[9.2–45.2]
**Recurrence volume** (**cm**^3^)				
Average/stdev	13.9 ± 19.7	22.9 ± 24.0	20.3 ± 26.7	8.6 ± 11.9
Median	5.6	15.0	8.5	4.1
IQR	[1.7–17.7]	[5.6–31.9]	[2.0–26.7]	[1.3–10.2]
**Center of Mass Distance (cm)**				
Average/stdev	2.2 ± 1.9	1.7 ± 1.5	3.0 ± 2.8	1.9 ± 1.4
Median	1.6	1.4	2.1	1.6
IQR	[0.9–2.6]	[0.8–2.1]	[1.2–3.4]	[0.9–2.5]
**Age (years)**				
Average/stdev	62.4 ± 10.2	63.0 ± 9.2	62.1 ± 9.6	62.4 ± 10.8
**OS (months)**				
Average/stdev	16.6 ± 9.3	14.4 ± 8.9	20.9 ± 10.1	15.4 ± 8.6
**Time to recurrence (months)**				
Average/stdev	11.2 ± 9. 6^†^	8.9 ± 6.5	16.1 ± 12.4	9.0 ± 7. 2^†^
**Extent of resection**				
Gross-total resection ( > 95%)	43% (104)	100% (40)	0	45% (64)
Near-total resection ( > 90%)	8% (19)	0	0	13% (19)
Subtotal resection ( < 90%)	5% (11)	0	0	8% (11)
N/A	45% (109)	0	100% (61)	34% (48)
**Diagnosis**				
WHO grade 4 glioma	100% (243)	100% (40)	100% (61)	100% (142)
IDH Wildtype	98.4% (239)	90% (36)	100% (61)	100% (142)
IDH Mutant	1.6% (4)	10% (4)	0% (0)	0% (0)

^†^Statistic unavailable for 48 cases.

### Recurrence coverage

**Fig. 1:**
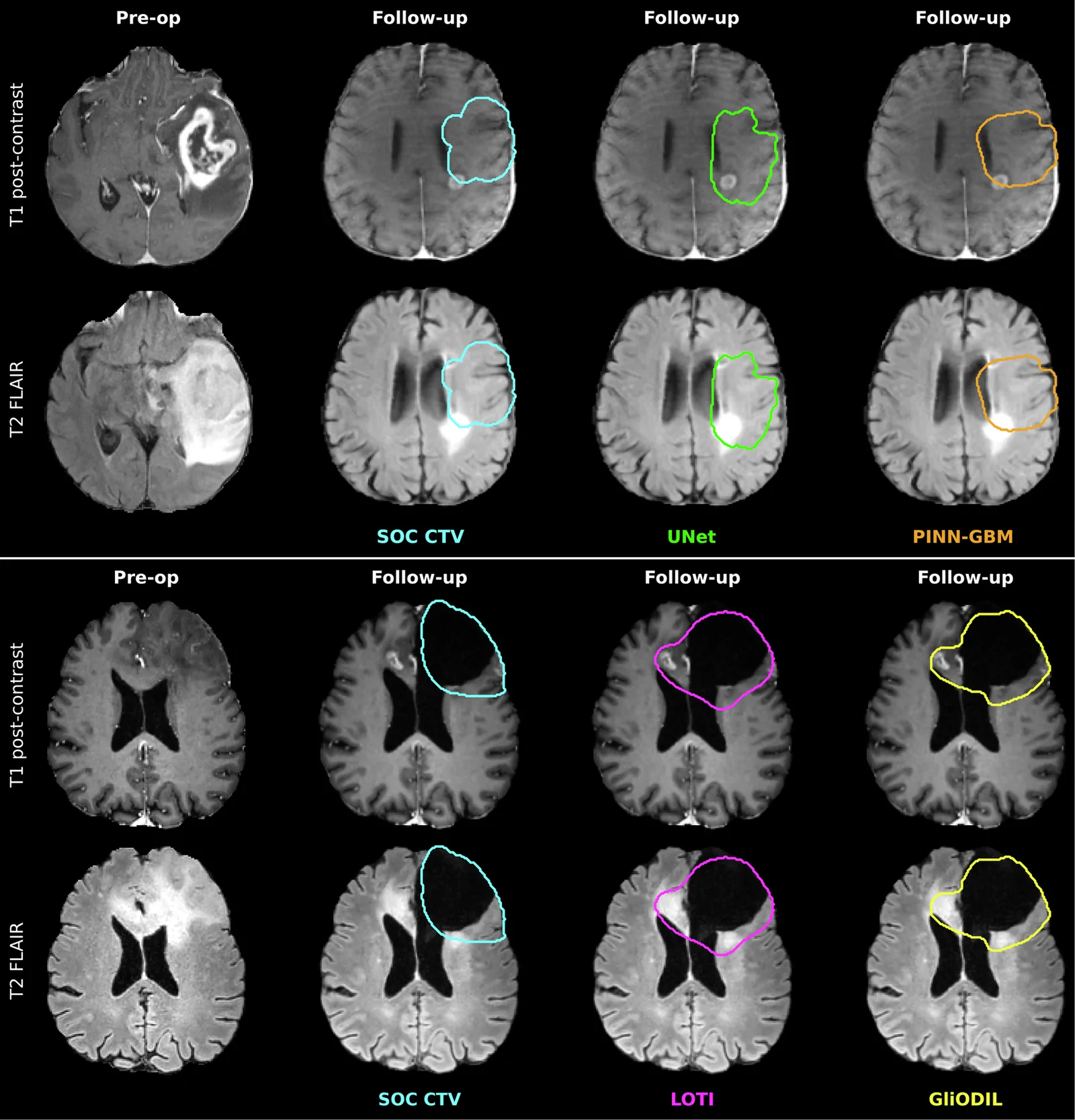
Comparison of CTVs for 2 example patients. Axial slices are centered on the pre-operative tumor center of mass for pre-op and on the recurrence center of mass for follow-up exams. Follow-up images are deformably registered to the pre-operative space. CTVs, shown as contours, are iso-volumetric: SOC CTV (cyan), U-Net CTV (lime), PINN-GBM CTV (orange), LOTI CTV (magenta), and GliODIL CTV (yellow). While the SOC CTV misses significant portions of the recurrent tumor, the model-based CTVs redistribute volume to cover the recurrence, while simultaneously shifting dose away from non-recurring tissue.

Model performance was evaluated for a wide variety of models, spanning both learning- and modeling-based approaches as well as their combination, using two clinically relevant recurrence definitions: (i) enhancing recurrence (Table 2a), and (ii) enhancing plus necrotic recurrence (Table 2b). Results are reported for each individual contributing dataset and for the aggregated PREDICT-GBM cohort, with representative qualitative examples shown in Fig. 1. We also report results for the subset of patients with available diffusion images, as this modality is required for GlioMap (excluding RHUH, which was included in the GlioMap training). Please note that all plans are *iso-volumetric* to the guideline-based comparator to allow for an unbiased, fair comparison. Across the combined cohort, our newly developed U-Net achieved the highest mean coverage for both recurrence definitions, closely followed by GliODIL. The U-Net, GliODIL, Lightweight Optimization for Estimating Tumor Infiltration (LOTI), PINN-GBM, and GlioMap all performed at least as well as the guideline-based comparator. In contrast, both Learn-Morph-Infer (LMI) and the nnU-Net baseline underperformed by approximately 10% and 20%, respectively, across the aggregated cohort. Including necrosis in the recurrence definition produced only modest changes in mean coverage compared with an enhancing-only evaluation.

**Table 2:** Coverage and standard error for (a) enhancing recurrence, and (b) recurrence core (enhancing + necrotic)

(a) Enhancing recurrence								
Dataset	SOC	LOTI	GliODIL	U-Net	PINN-GBM	LMI	nnU-Net	GlioMap
RHUH	82.64 ± 4.26	82.38 ± 3.97	83.64 ± 4.16	**85.26** ± **3.97**	83.17 ± 4.17	70.31 ± 4.00	53.69 ± 5.12	−
TUM-GBM	79.89 ± 2.81	81.88 ± 2.58	**82.13** ± 2.61*	81.80 ± 2.66*	80.84 ± 2.78	68.48 ± 2.92	60.57 ± 2.71	−
LUMIERE	67.91 ± 4.76	68.26 ± 4.56	68.29 ± 4.70	**69.87** ± 4.72*	67.98 ± 4.78	62.31 ± 4.62	59.29 ± 4.89	−
DIFFUSION	82.73 ± 2.84	**86.12** ± 2.40*	85.38 ± 2.63*	84.63 ± 2.70*	83.41 ± 2.86	71.37 ± 3.04	62.51 ± 3.15	83.73 ± 2.53
PREDICT-GBM	77.34 ± 2.17	78.54 ± 2.03	78.91 ± 2.08*	**79.37** ± 2.08*	77.99 ± 2.16	67.23 ± 2.10	59.12 ± 2.45	−
*p*_PREDICT_	−	0.46	1.0 × 10^−3^	2.9 × 10^−5^	0.10	1.00	1.00	−

(b) Recurrence core (enhancing, necrotic)								
RHUH	82.93 ± 4.25	82.76 ± 3.97	83.99 ± 4.15	**85.54** ± **3.97**	83.44 ± 4.17	70.60 ± 4.02	53.91 ± 5.13	−
TUM-GBM	79.93 ± 2.80	82.04 ± 2.57	**82.27** ± 2.61*	81.90 ± 2.65*	81.01 ± 2.78	68.35 ± 2.91	60.69 ± 2.67	−
LUMIERE	67.78 ± 4.79	68.17 ± 4.59	68.15 ± 4.74	**69.74** ± **4.76**	67.81 ± 4.81	62.23 ± 4.65	59.01 ± 4.91	−
DIFFUSION	82.75 ± 2.83	**86.26** ± 2.39*	85.52 ± 2.62*	84.73 ± 2.69*	83.59 ± 2.85	71.38 ± 3.03	62.53 ± 3.14	83.76 ± 2.52
PREDICT-GBM	77.37 ± 2.17	78.68 ± 2.03	79.01 ± 2.08*	**79.44** ± 2.09*	78.09 ± 2.16	67.18 ± 2.17	59.15 ± 2.45	−
*p*_PREDICT_	−	0.42	1.0 × 10^−3^	2.7 × 10^−5^	0.08	1.00	1.00	−

*indicates *p* < 0.05 (paired Wilcoxon signed-rank test, Benjamini-Hochberg adjusted) comparing model plans to the guideline-based comparator (SOC^8^, a simplified 15-mm geometric expansion, not a clinical treatment plan); **bold** indicates best value per dataset. *p*_PREDICT_: comparison against SOC in the full PREDICT-GBM dataset (*N* = 243). DIFFUSION: subset of PREDICT-GBM with ADC/DTI images (*N* = 112).

Although the primary evaluations were performed in pre-operative reference space, clinical radiotherapy target delineation is performed on post-operative imaging. We therefore conducted an additional post-operative space sensitivity analysis to assess whether the comparative model rankings were preserved after transfer into post-operative anatomy (Supplementary Fig. 3). The relative ordering of methods remained unchanged, suggesting that the benchmark findings are robust to the choice of anatomical reference space. This analysis remains geometric, however, and should not be interpreted as a full post-operative radiotherapy planning evaluation.

To assess robustness to inter-center domain shift, we retrained the U-Net in a leave-one-dataset-out configuration, holding out RHUH entirely as the test set. Mean coverage decreased by 1.0% for the enhancing recurrence and 1.02% for the enhancing+necrotic recurrence, indicating modest degradation under center-level distribution shift.

To isolate the contribution of the extended training cohort and to provide a matched-input comparison against the nnU-Net baseline, we ran two additional ablations. (i) Retraining the U-Net ensemble on only the three datasets comprising PREDICT-GBM yielded 79.26 ± 2.02% coverage of the enhancing recurrence, confirming a minor gain from the additional 84 training cases. (ii) Restricting the inputs to those used by the nnU-Net baseline (tissue maps and tumor segmentation) and training without ensembling on the same three datasets yielded 78.86 ± 2.03%, indicating a minor improvement from the additional input modalities. Ablating the distance-based post-processing reduced coverage by 0.67% for the enhancing recurrence and 0.73% for the enhancing+necrotic recurrence; the alternative white-matter weighted prior reduced coverage further ( − 0.72% at w_WM_ = 0.2, − 14.7% at w_WM_ = 0.5).

The Dice coefficient ranked the U-Net first (mean 10.67 ± 0.78 versus 10.34 ± 0.77 for SOC; Supplementary Fig. 1), while SOC achieved the best median 95% Hausdorff distance (32.02 mm; PINN-GBM best mean at 36.44 ± 1.21 mm; Supplementary Fig. 2). Further details are given in Supplementary Note 1.

### Statistical analysis

**Fig. 2:**
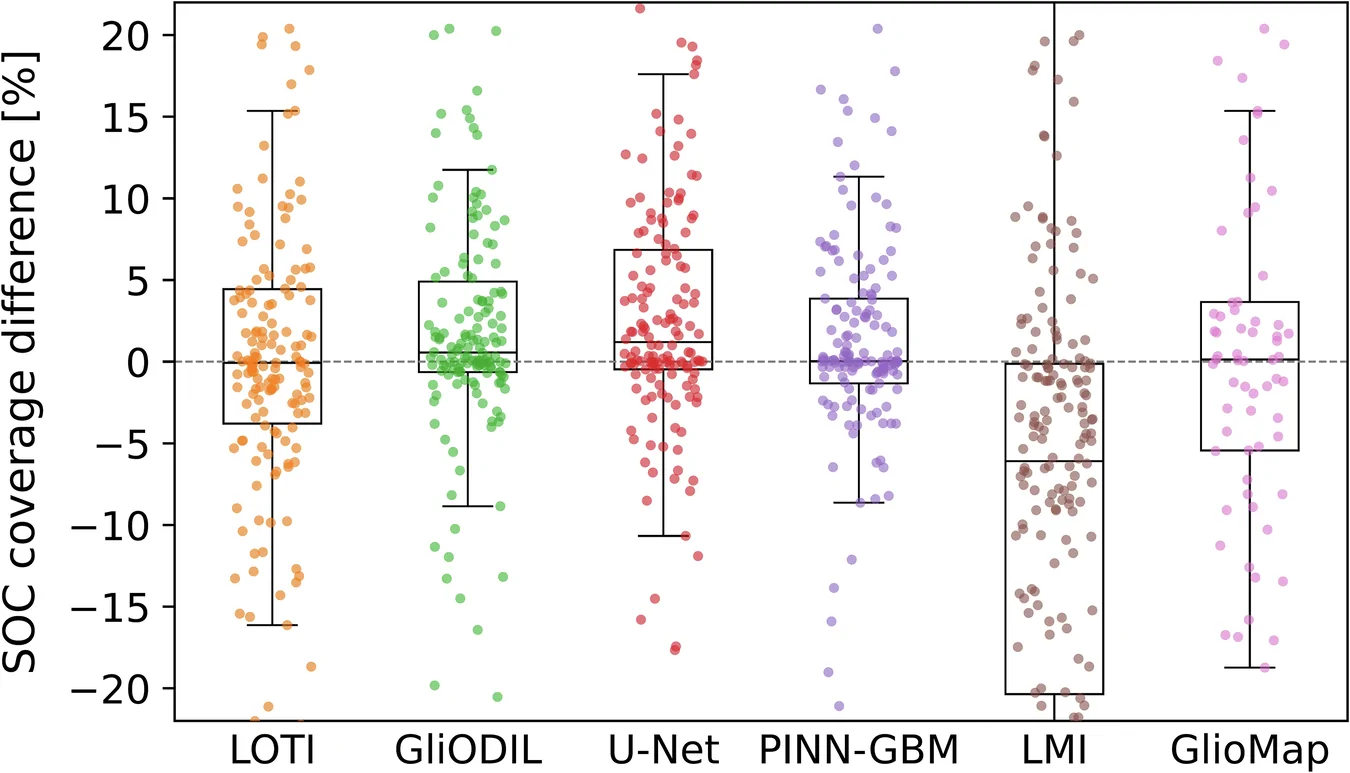
Differences between model coverage and SOC coverage. Per-patient differences between model coverage and SOC coverage, excluding cases in which model and SOC achieved the exact same coverage. Positive values indicate a higher model coverage compared to SOC. We note that ties (identical coverage) are frequent under often saturated coverages and iso-volumetric constraint, and are excluded here for visual clarity.

Both our U-Net and GliODIL showed significant improvements in enhancing-only and enhancing + necrosis recurrence on the PREDICT-GBM cohort (*p* < 10^−4^ and *p* ≤ 10^−3^, respectively). At the dataset level, our U-Net and GliODIL achieved significant (*p* < 0.05) improvement over the guideline-based comparator on TUM-GBM, and, with LOTI, the diffusion subset. To further investigate spatial factors associated with model performance, we analyzed the relationship between recurrence location and the improvement in coverage over the comparator CTV. Using Spearman rank correlation *ρ*, we observed a weak positive correlation (*ρ* between 0.2 and 0.3) between CoM distance and improvement over the guideline-based comparator within the clinically relevant CoM distance range of 15 − 30 mm for our U-Net, GliODIL and LOTI. This suggests that model-based CTVs tend to provide greater benefit for recurrences occurring at intermediate distances from the primary tumor, a spatial range that may be particularly challenging for uniform margin-based expansions. PINN-GBM achieved the best median coverage across the combined cohort (97.8%), while our U-Net achieved the best median coverages for the individual datasets. The U-Net also achieved the lowest IQR on the combined dataset (Supplementary Table 1). Figure 2 illustrates the per-patient improvement in recurrence coverage relative to SOC excluding ties (identical coverage) for better visualization. Paired effect sizes summarized by the Hodges–Lehmann estimator, estimated on the subset of patients whose model and SOC coverages differ (tied paired differences excluded), rank the U-Net first and GliODIL second on the combined cohort (U-Net + 2.23%, 95% CI [1.15, 3.56]; GliODIL + 1.27% [0.47, 2.35]; Supplementary Table 1). These shifts therefore quantify the paired effect within the subset and should not be read as full-cohort mean shifts. At least a 5% absolute improvement over the SOC plan is observed in 19.3% of patients for the U-Net and 16.5% for GliODIL. Subgroup analyses stratified by primary tumor volume, recurrence center-of-mass distance, and number of tumor or recurrence foci are reported in Supplementary Fig. 4. Coverage of model-based plans improves slightly over SOC for recurrences at intermediate center-of-mass distances, consistent with the Spearman analysis above. Further details are given in Supplementary Note 2.

### Sensitivity to longitudinal registration and segmentation

**Fig. 3:**
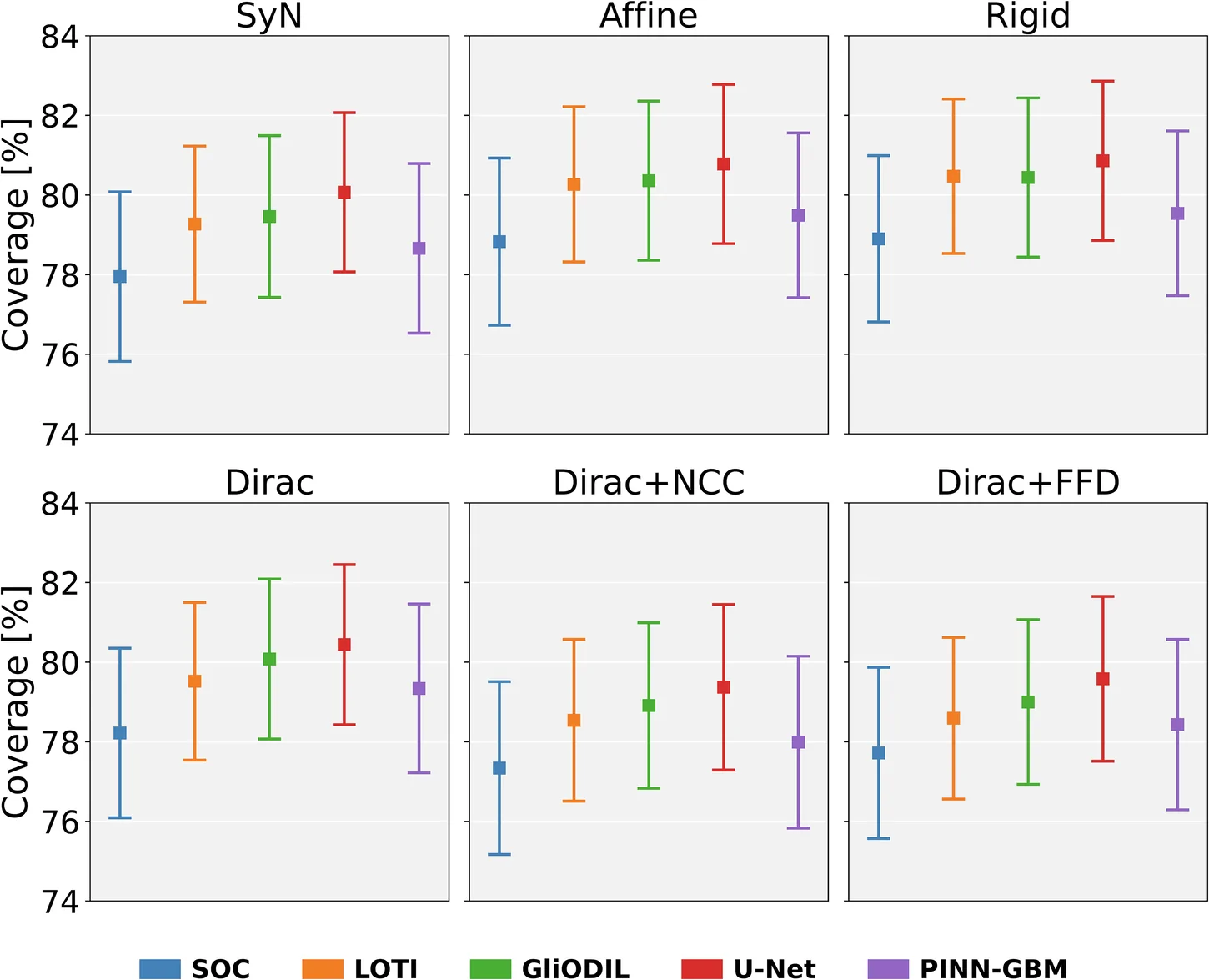
Recurrence coverage across different longitudinal registration algorithms. Coverage of enhancing recurrence for the combined cohort using different longitudinal registration algorithms to map the follow-up exam from atlas space to the pre-operative exam. Error bars denote the standard error of the mean.

**Fig. 4:**
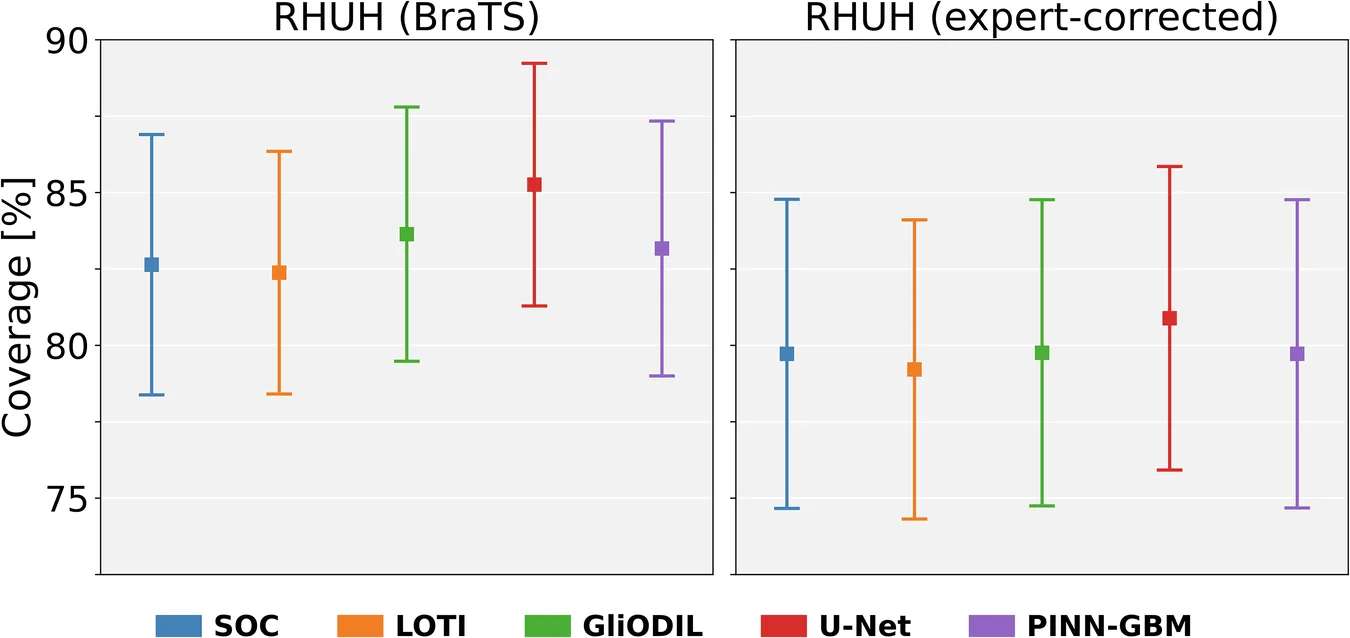
Recurrence coverage with RHUH expert-corrected segmentations. Coverage of enhancing recurrence on RHUH with recurrence segmentations from the BraTS 2023 winning algorithm^55^ and expert-corrected segmentations. Error bars denote the standard error of the mean.

Accurate evaluation of recurrence coverage requires voxel-wise correspondence between the pre-operative and follow-up exams. Longitudinal alignment in glioblastoma is challenging due to mass effect, post-operative changes, and therapy-related anatomical deformation. To assess the sensitivity of our results to the choice of longitudinal registration, we compared rigid and affine registration with deformable ANTs symmetric normalization (SyN)^51^ as well as the 2022 MICCAI BraTSReg challenge winning algorithm (Dirac+NCC)^52–54^ and an alternative instance optimization (Dirac+FFD) for mapping the follow-up exam into the pre-operative reference space. Figure 3 reports enhancing-recurrence coverage for each registration method. While absolute coverage values varied slightly across algorithms, the relative ranking of planning approaches remained unchanged. Consistently, conclusions from paired Wilcoxon signed-rank tests were stable across registration choices, indicating that the reported statistical findings are not driven by the specific longitudinal registration method, but are robust. Likewise, to assess the sensitivity of our results to the follow-up segmentation, we re-evaluated recurrence coverage on the RHUH cohort (*N* = 40) using the expert-corrected recurrence segmentations released with the dataset^49^ in place of the BraTS-derived follow-up segmentations used elsewhere in this study^55^. Only the evaluation ground truth was substituted; the models were neither retrained nor re-run with modified pre-operative inputs. Results are shown in Fig. 4. As with the registration sensitivity analysis, the relative ranking of planning approaches was preserved. However, absolute coverage values were systematically lower under the expert-corrected ground truth, and the gap between model-based plans and SOC narrowed substantially (SOC: 79.24 ± 5.05%, U-Net: 80.47 ± 4.95%, GliODIL: 79.23 ± 5.01%). We therefore regard this as a reassuring but limited sensitivity analysis rather than strong evidence of robustness. The broad ranking is preserved, but the magnitude of the model advantage is sensitive to recurrence-annotation quality.

### U-Net predictions

**Fig. 5:**
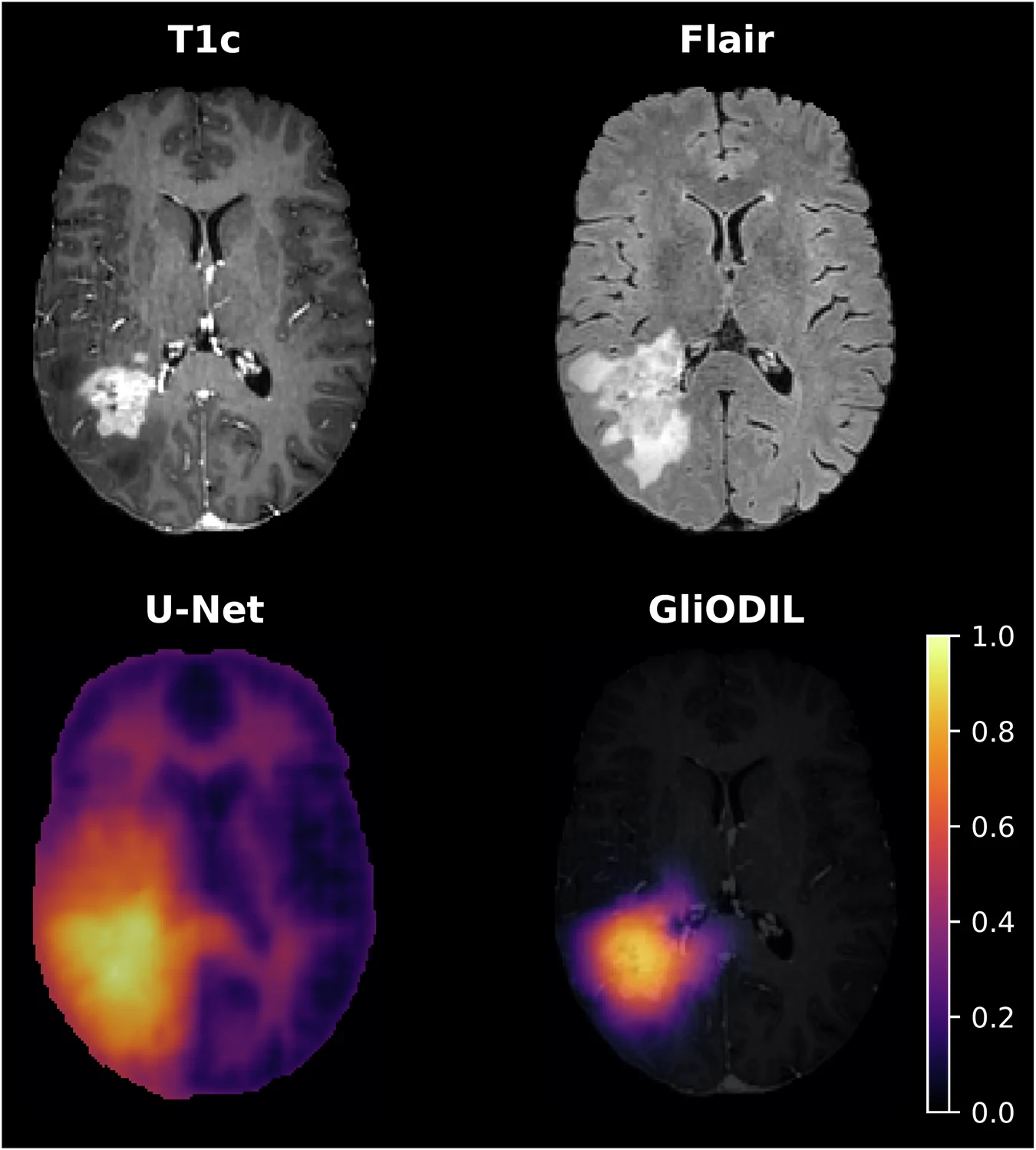
Representative model predictions. Representative outputs of our U-Net and GliODIL, showing predicted probability of future recurrence and estimated tumor cell concentration, respectively.

An example prediction is illustrated in Fig. 5. Qualitative analysis indicates that white matter regions are assigned a higher probability of recurrence, while gray matter and CSF are assigned a lower probability. This is consistent with the higher diffusivity in white matter and with biological and clinical studies on glioblastoma recurrence^56,57^. Additionally, cell migration from one hemisphere to the other is predicted mostly through the corpus callosum, indicating that the model does not only predict via proximity but indeed learns meaningful, biologically plausible spatial patterns.

### Failure cases

**Fig. 6:**
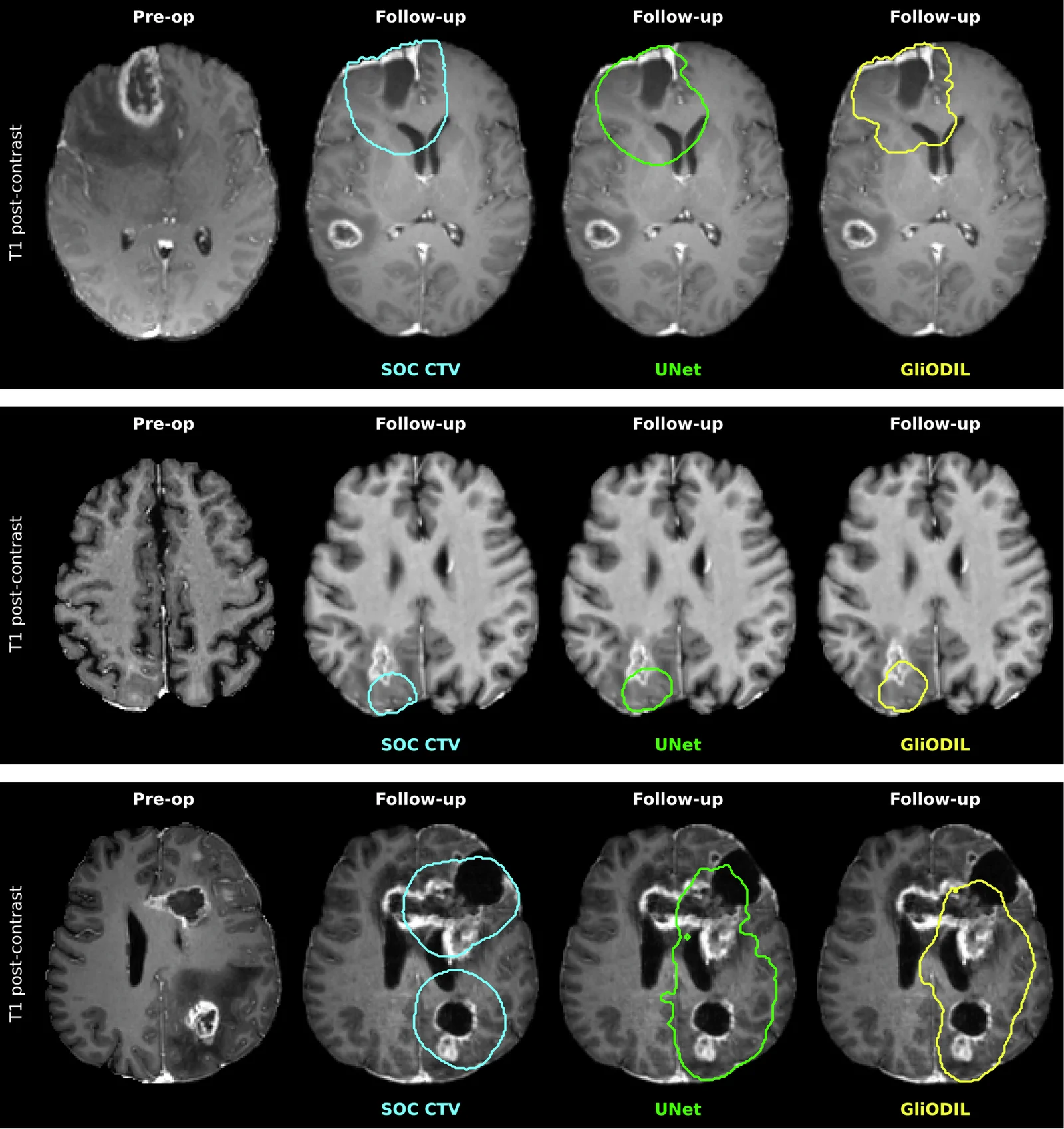
Comparison of CTVs for failure cases. Axial slices are centered on the pre-operative tumor center of mass for pre-op and on the recurrence center of mass for follow-up exams. Follow-up images are deformably registered to the pre-operative space. CTVs, shown as contours, are iso-volumetric: SOC CTV (cyan), U-Net CTV (lime), and GliODIL CTV (yellow). The top case illustrates a distant recurrence, the middle case a small primary tumor with a comparatively large recurrence, and the bottom case a multifocal tumor.

Representative cases with particularly low recurrence coverage are shown in Fig. 6. Distant recurrences were not adequately captured by either the guideline-based CTV or the model-based CTVs. In cases with very small initial tumors, the limited baseline volume constrains iso-volumetric redistribution, resulting in near-spherical CTVs that fail to extend toward the eventual recurrence site. For multifocal tumors, the SOC outperformed model-based approaches, as physics-informed models typically assume a single initial tumor position and therefore do not explicitly account for multifocal disease, whereas data-driven models suffer from underrepresentation of multifocal tumors in the training data.

## Discussion

Although advances in radiotherapy delivery have enabled highly conformal dose distributions, current glioblastoma target definition still largely relies on isotropic margin expansions around MRI-visible disease, without accounting for spatially heterogeneous invasion patterns, patient-specific tumor biology, or anatomical constraints. This paradigm risks undertreating infiltrative tumor that extends beyond the chosen margin, as shown by previous research^8,11,13,14,20^, while simultaneously irradiating substantial volumes of normal brain tissue, with negative implications for cognitive function, quality of life, and even survival^58^. Computational growth models offer an appealing alternative: by estimating tumor cell density beyond the enhancing lesion and edema, they could support biologically informed and individualized target volumes. Furthermore, purely data-driven models could leverage advancements in machine learning for recurrence prediction, a task conceptually distinct from glioblastoma growth modeling and that does not explicitly estimate underlying tumor cell dynamics. However, progress toward clinical translation has been constrained by limited access to suitable longitudinal imaging, heterogeneous preprocessing choices, and inconsistent evaluation protocols, making it difficult to determine when model-based plans truly improve upon standard practice. This study addresses these barriers by introducing PREDICT-GBM as a standardized platform for glioblastoma growth modeling in the context of radiotherapy planning. PREDICT-GBM provides (i) a large, expert-curated, longitudinal multi-center dataset, (ii) an end-to-end open-source pipeline spanning preprocessing, segmentation, registration, model inference, and plan evaluation, and (iii) a containerized collection of growth and data-driven models that can be readily executed within the pipeline and easily extended to incorporate new modeling approaches. By providing both processed inputs and an extensible framework, PREDICT-GBM is intended to reduce methodological variability and shift the emphasis toward clinically relevant comparisons across heterogeneous cohorts, which are critical for future clinical translation. Importantly, our results suggest that, in this retrospective evaluation, both data-driven recurrence-prediction models and biophysical growth models can achieve higher geometric coverage of later recurrence regions than guideline-based comparator plans. This stringent evaluation of parallel approaches allows us to draw meaningful conclusions and outline promising future research directions: (i) expanding and enriching clinical datasets through multi-center data sharing and the inclusion of spatially resolved biopsy data to improve biological calibration and validation and to improve the performance of data-driven models, (ii) advancing physics-driven modeling beyond classical diffusion-reaction formulations by incorporating mechanisms such as mass effect, multi-compartment tumor representations, and explicit handling of multifocal disease, as well as integrating additional modalities including diffusion, perfusion, or PET imaging, and (iii) moving beyond purely geometric target comparisons toward dose-aware evaluation frameworks, where model-derived risk maps can guide dose painting or boosting while respecting organ-at-risk constraints, ultimately paving the way to clinical adoption of computational models for individualizing radiotherapy planning.

Across the aggregated cohort, for both the enhancing and enhancing-plus-necrotic recurrence definitions, our U-Net achieved the highest mean recurrence coverage, followed by GliODIL, LOTI and PINN-GBM, each of which outperformed the guideline-based comparator. GlioMap likewise exceeded SOC on the subset of patients with available diffusion imaging. LMI (trained on synthetic data) and the nnU-Net baseline produced lower mean coverage than the guideline-based comparator. Importantly, the U-Net and GliODIL demonstrated a statistically significant improvement over the guideline-based comparator for both enhancing-only recurrence and enhancing-plus-necrotic recurrence in the pooled cohort (paired Wilcoxon signed-rank tests). Beyond mean coverage, PINN-GBM achieved the best median coverage across the combined cohort, while the U-Net achieved the best median coverage per dataset and the lowest interquartile range across the combined cohort, indicating more consistent patient-level performance. Paired effect sizes, summarized by the Hodges-Lehmann estimator, ranked the U-Net first and GliODIL second on the combined cohort. Only the U-Net and GliODIL reached pooled-cohort significance for enhancing-only definitions over SOC. PINN-GBM, despite its strong combined-cohort median, did not, underscoring that aggregate coverage improvements alone do not guarantee consistent patient-level benefit. Adding necrosis to the enhancing recurrence definition produced only modest changes in mean coverage, consistent with necrosis often co-localizing with enhancing progression and therefore not substantially altering the spatial evaluation target. Because model-based targets were constrained to be iso-volumetric with the SOC plan, any increase in recurrence coverage necessarily corresponds to a matched decrease in volume placed outside the eventual recurrence. Our results, therefore, show that, in this retrospective geometric setting, model-based target volumes can shift coverage toward regions of later recurrence at a fixed treatment volume. Whether such geometric shifts translate into improved survival, reduced toxicity, or improved quality of life cannot be inferred from this analysis and would require dose-aware planning, organ-at-risk evaluation, and prospective or outcome-based validation.

Further analysis of spatial patterns revealed a weak positive correlation between CoM distance and coverage improvement over the comparator CTV within the clinically relevant 15 − 30 mm range. This finding suggests that model-based approaches may be particularly beneficial for intermediate distances, beyond the typical high-density tumor core but within a biologically plausible infiltration range, where uniform margin expansions may be suboptimal. Finally, sensitivity analyses of the longitudinal registration step demonstrated that, while absolute coverage values varied slightly across registration algorithms, the relative ranking of methods remained unchanged, and statistical significance conclusions were robust across registration strategies. Likewise, a sensitivity analysis on the RHUH cohort using the expert-corrected follow-up segmentations in place of the BraTS-derived ground truth preserved the relative ranking of methods, though absolute coverage values were systematically lower and the gap between model-based plans and SOC narrowed. This constitutes a reassuring but limited sensitivity analysis rather than strong evidence of robustness. Although the ranking is preserved, the absolute model advantage is largely reduced under expert-corrected annotations. A more conclusive evaluation would require a larger expert-annotated cohort and full training and evaluation on expert-corrected segmentations.

A key challenge in comparative evaluation for recurrence prediction is the requirement for high-quality, disease-specific preprocessing. Model performance depends on the quality of upstream preprocessing, and end-to-end evaluation necessitates careful orchestration of numerous components, which in turn require domain expertise that is not universally available, particularly outside specialized research centers. Evaluation requires atlas registration and longitudinal registration, both robust to mass effect and postsurgical deformation, as even small misregistrations can meaningfully distort voxel-level overlap metrics, especially for small recurrences. Tumor segmentation is another necessary component of such pipelines and is particularly challenging in the presence of edema, resection cavities, and post-operative changes. High-quality delineations and quality control are difficult to ensure when medical expertise is not readily available. In addition, many growth models require tissue classification of the entire brain to modulate tumor cell migration according to the underlying tissue class. This segmentation is especially challenging in the tumor region, since images of the healthy brain before disease onset are typically unavailable. By making the full end-to-end pipeline open source, we aim to lower these barriers and make rigorous comparative evaluation more approachable and reproducible for the broader community.

A further challenge for benchmarking recurrence prediction in a radiotherapy context is the choice of evaluation metrics. For instance, a framework based solely on sensitivity and specificity is insufficient. Because the class imbalance between normal tissue and tumor is high, simply increasing the predicted target volume can substantially boost sensitivity while only marginally reducing specificity. We therefore require target volumes to be iso-volumetric, forcing models to redistribute a fixed treatment volume toward regions that increase both sensitivity and specificity, rather than benefiting from trivial volume inflation. The iso-volumetric constraint also matches the prevailing convention in the glioblastoma growth-model literature^12,20,30,32^, where pre-operative imaging is used to derive risk maps. With this constraint, the percentage-based definition of recurrence coverage is equivalent to the sensitivity, since the true positives correspond to the overlap between the target volume and recurrence, and the sum of true positives and false negatives equals the recurrence volume. Clinical radiotherapy planning, by contrast, is based on post-operative anatomy, residual enhancement, and cavity geometry^8^. To assess the suitability of risk maps derived from pre-operative imaging for post-operative radiotherapy planning, we re-evaluated all models in post-operative space and found that the relative ordering of methods is preserved. In a prospective workflow, the pre-operative risk map could therefore be deformably registered to the post-operative planning MRI and provide auxiliary information for target definition by a clinician. Nonetheless, this geometric evaluation does not capture dose falloff, dose painting/boosting strategies, or organ-at-risk constraints. A more complete clinical validation would require training and evaluating models directly in the post-operative space and performing full dosimetric, organ-at-risk-aware treatment planning analyses. We therefore regard the present benchmark as an intermediate step toward such future dose-aware studies.

Several limitations of this study should be acknowledged. Although our cohort size is large relative to prior studies, it remains modest compared with typical deep learning requirements, particularly under multi-center domain shift and heterogeneous acquisition protocols. Continued data collection and responsible data sharing by medical institutions are essential to enable larger, more representative cohorts and more definitive comparative evaluations. Additionally, the current benchmark is constrained to routinely acquired standard MRI (with ADC only available for a subset), and does not yet incorporate modalities such as PET, diffusion tensor imaging, perfusion, or MR spectroscopy, which may provide complementary biological information and have been reported to improve model predictions in related work^31,32,36^. Although integration of additional modalities into PREDICT-GBM is straightforward, there is currently insufficient longitudinal data including these modalities. Evaluation is also limited by the quality of the longitudinal registration and the tumor segmentations. Our framework uses the 2022 MICCAI BraTSReg challenge winning algorithm, which achieved a median absolute error of 1.64 mm ^54^, and the BraTS 2023 / 2024 winning segmentation methods^41,55^. To investigate the impact of these upstream uncertainties on our result, we compared four longitudinal registration algorithms (Fig. 3) and evaluated coverage on RHUH using the expert-corrected follow-up segmentations released with that dataset (Fig. 4). In both analyses, the relative ranking of planning approaches was preserved, although the gap between model-based plans and SOC narrowed under expert-corrected recurrence segmentations. Although this evaluation did not re-evaluate or re-train models with expert-corrected pre-operative segmentations, these results motivate further investment in expert-validated tumor segmentations. The benchmark also evaluates geometric target definitions rather than delivered dose distributions. Therefore, the observed improvement in recurrence coverage cannot be directly interpreted as expected gains in survival, reduced toxicity, or improved quality of life. Ultimately, clinical impact will require validation and integration into treatment planning workflows, which may include strategies such as dose painting, boosting, or other dose-aware adaptations within model-predicted high-risk regions. Moreover, the follow-up MRIs used for evaluation reflect outcomes under standard-of-care treatment. Consequently, observed recurrence patterns can be interpreted as a spatial proxy for regions of elevated residual tumor burden. In this context, predicting the recurrence core corresponds to identifying high-risk areas that were insufficiently treated by uniform margin-based irradiation, providing a clinically meaningful target. However, this retrospective evaluation should not be conflated with prospective assessment of fully model-based radiation plans, where altered dose distributions may lead to different recurrence patterns and would require dedicated clinical validation. Finally, model validation is limited by the lack of ground truth in non-MRI-visible tumor regions. This could be addressed by incorporating spatially resolved biopsy data obtained during surgery, enabling calibration of predicted cell density maps beyond the imaging-defined lesion. As a platform, PREDICT-GBM is intentionally designed to be extensible, allowing new datasets, modalities, models, registration backends, and evaluation metrics to be added with ease. However, the current release is constrained by the modalities and labels available in its source cohorts (T1, T1c, T2, FLAIR, ADC, PET). Perfusion, MR spectroscopy, or dose maps are not yet part of the platform and remain natural next additions.

Looking forward, PREDICT-GBM is designed to accelerate model development and translation by enabling standardized, reproducible evaluation on any suitable cohort. We release anonymized longitudinal MRI data for 142 patients, thereby increasing the availability of public datasets suitable for growth modeling and recurrence prediction research. We hope that in the future, more medical institutions will consider releasing their data, as this is essential for enabling translational research. Beyond increasing MRI cohort sizes, integrating spatially resolved biopsy data could be invaluable for calibrating growth models and for more precisely defining tumors and recurrences within and beyond the MRI-visible region. The two model families benchmarked here have complementary strengths and limitations. Biophysical reaction-diffusion models yield interpretable, patient-specific tumor-cell density maps and propagate well to settings with little training data, but depend on the underlying PDE capturing the relevant tumor dynamics. Data-driven recurrence predictors can capture statistical patterns, including non-physical ones, and can offer sub-second inference, but depend heavily on the composition of the training cohort and provide limited mechanistic interpretability. Methodologically, future growth models could therefore incorporate biophysical mechanisms beyond diffusion-reaction frameworks. For instance, integrating mechanistic mass effect could yield better estimates of tumor cell concentration, particularly for large tumors. Multi-compartment models that distinguish among tumor cell types, such as hypoxic, proliferative, or invasive, could better capture the heterogeneous nature of glioblastoma. In addition, extending physics-informed models to explicitly account for multifocal disease, via multiple tumor seeds or more flexible initialization strategies that allow spatially separated baseline tumor regions, could improve their applicability to patients with multifocal tumors. Our platform naturally supports extending preprocessing to additional modalities suitable for such models, such as diffusion tensor imaging, perfusion, or PET. Diffusion imaging could yield information about local, anisotropic diffusivity beyond the typical fixed values assumed for gray and white brain matter, while perfusion imaging provides information about oxygenation. PET, which indicates regions of metabolic activity, in particular, has been shown to significantly benefit growth models, although available data is scarce^31,32^. In general, growth models scale with the number of modalities, as each provides additional information about the underlying biophysical processes. Highly data-driven models, such as U-Nets, scale with the number of subjects, underscoring the need for further data sharing. Finally, future evaluations should move beyond geometric targets toward dose-aware planning: model-derived cell density or risk maps could guide dose painting or boosting, delivering higher doses to regions of high predicted tumor cell concentration while respecting organ-at-risk constraints and realistic dose falloff.

PREDICT-GBM provides a standardized, open-source framework for benchmarking and developing models for personalized glioblastoma radiotherapy planning, as demonstrated by our U-Net. By unifying data processing and providing reproducible benchmarks, PREDICT-GBM lowers the barrier to entry for researchers in this area. Our results show that, under iso-volumetric constraints and retrospective evaluation, both physics-informed and data-driven models can yield radiotherapy target volumes with statistically significant gains in geometric coverage of regions of later recurrence relative to guideline-based comparator plans. With our framework, we hope to foster research in both directions and to help build the necessary evidence base for future clinical validation of computational growth models for personalized radiotherapy planning.

## Methods

### Pipeline

**Fig. 7:**
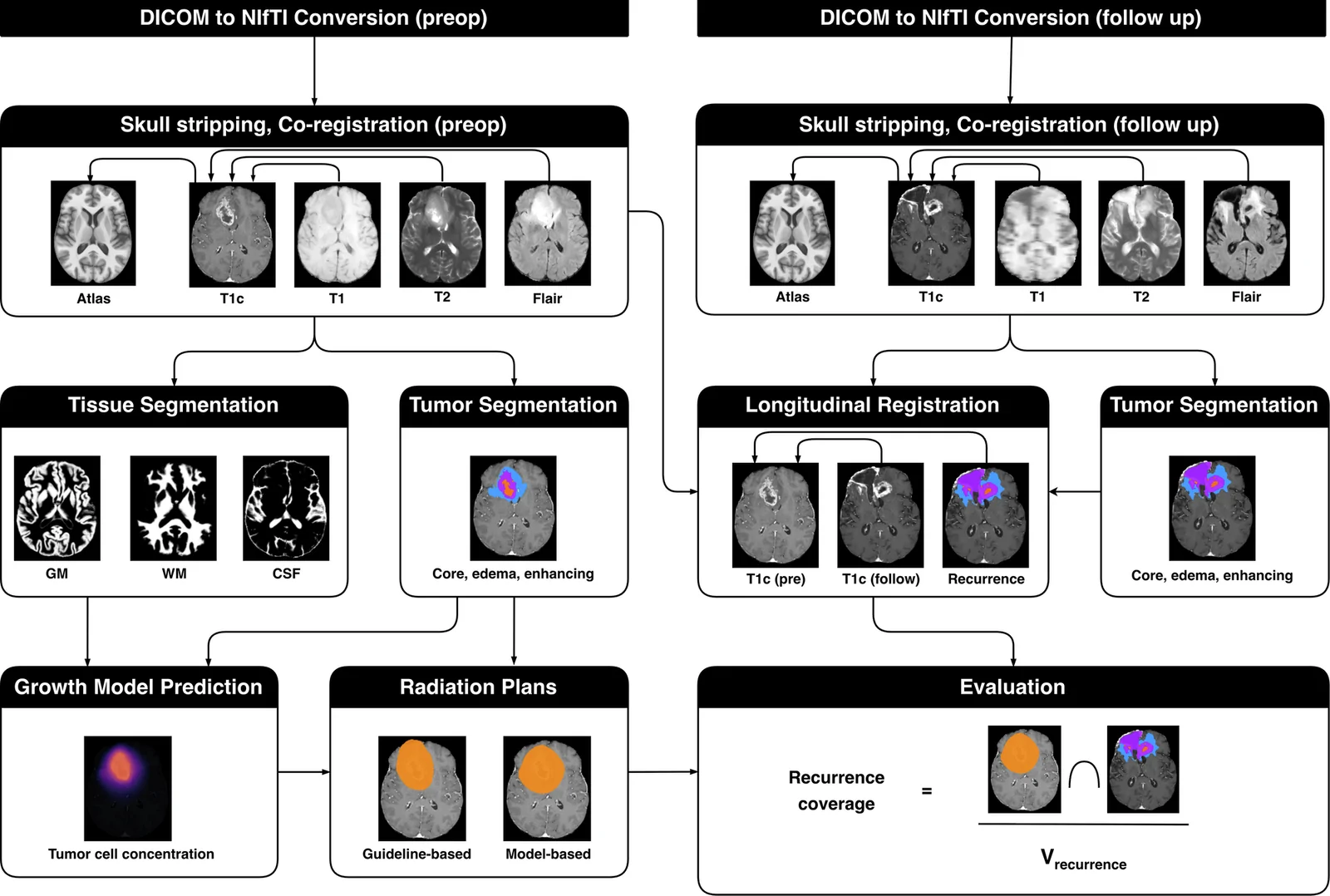
Visualization of the PREDICT-GBM pipeline. After converting DICOM inputs to NIfTI format, the skull is stripped, and the modalities are co-registered to the SRI24 atlas space for both the pre-operative and follow-up exams. The tumor is then segmented into enhancing tumor, necrotic tumor, and peritumoral edema for both exams. For the pre-operative exam, the brain tissue is further segmented into white matter, gray matter, and cerebrospinal fluid. The pre-operative tumor and tissue segmentations are then used as input for the models. The follow-up segmentation is deformably registered to the pre-operative exam. Lastly, radiation plans (incl. SOC) are generated, and their coverage is computed on the registered recurrence.

Evaluating tumor growth models for radiation planning requires numerous processing steps. A schematic overview of the PREDICT-GBM pipeline is shown in Fig. 7. Tools and dependencies were chosen based on performance and runtime considerations, with a preference for open-source solutions. Likewise, our entire pipeline is open source, providing researchers with a plug-and-play framework for growth model evaluation (github.com/BrainLesion/PredictGBM). In the following, we describe the different modules of our pipeline in the order of execution.

### Inclusion criteria

Subjects were curated from three sources: LUMIERE^48^, RHUH^49^, and TUM-GBM^30,32^. The inclusion criteria required (i) a pre-operative MRI exam, (ii) at least one follow-up exam demonstrating early post-treatment recurrence, (iii) availability of standard MRI sequences for each exam (T1-weighted (T1), contrast-enhanced T1-weighted (T1c), T2-weighted (T2), and fluid-attenuated inversion recovery (FLAIR)). Follow-up exams were required to be acquired at least 12 weeks after the pre-operative baseline exam, as pseudo-progression is most commonly observed in these first weeks of treatment^59^. For patients with multiple follow-up exams, we identified the exam with the best resolution that met the other criteria for inclusion. To minimize site-specific preprocessing variability, we preferred the least-processed image format available (typically raw DICOM). All listed datasets were reviewed by an expert to confirm the presence of visible recurrence in the follow-up MRI scans.

### Image preprocessing

Images were converted from DICOM to NIfTI using dcm2niix^60^. All modalities were normalized and co-registered to the anatomical SRI24 atlas space^61^ using the BrainLes preprocessing module^42^. Within each exam, modalities were first rigidly registered to the T1c image and then co-registered to atlas space using mutual information as metric and the ANTs library as backend^62^. Skull stripping was performed with HD-BET^63^. For longitudinal alignment, the follow-up T1c was deformably registered to the pre-operative T1c using an implementation of the winning algorithm of the 2022 MICCAI BraTSReg challenge. This method uses a conditional deep Laplacian pyramid image registration network with a forward-backward consistency constraint, followed by a non-linear, multi-scale instance optimization using a normalized cross-correlation loss^54^. For comparison (see Fig. 3), we also implemented a SyN registration using ANTs SyN^51^, with optimal settings identified in the BraTSReg challenge^52^, as well as an alternative instance optimization minimizing a mean squared intensity error with a diffusion-style smoothness penalty. The resulting transforms were applied consistently to all follow-up modalities and derived segmentations to ensure voxel-wise correspondence in the pre-operative space. For patients with diffusion tensor imaging (DTI) as only diffusion modality, apparent diffusion coefficient (ADC) was derived via mean diffusivity (MD).

### Tumor segmentation

Tumor subregion segmentations were obtained from the preprocessed MRI using the BraTS pipeline^40^. Pre-operative segmentation used the BraTS 2023 winning method, an ensemble trained with heavy data augmentation ^55^. Follow-up segmentation used the BraTS 2024 winning method for post-operative glioma MRI^41^. Segmentations included necrosis, contrast-enhancing tumor, and peritumoral edema, enabling evaluation of recurrence under multiple clinically relevant definitions. The presence of a tumor was verified by a medical expert for all cases.

### Brain tissue segmentation

Several models require brain tissue segmentations to inform biophysical parameters, such as local diffusivity, which, in turn, affect cell migration. Because tumor and treatment effects disrupt local anatomy, we used an atlas-based approach to obtain tissue priors in patient space. Specifically, the SRI24 atlas was deformably registered to each subject using ANTs SyN^51^, and atlas tissue probability maps were warped into patient space using the obtained transformation. Tissue labels (gray matter, white matter, cerebrospinal fluid (CSF)) were derived from the warped probability maps and were used as model inputs where required.

### Models

We evaluated a diverse set of approaches spanning biophysical reaction-diffusion growth modeling and purely data-driven recurrence prediction. Models that estimate a tumor cell concentration map in our benchmark are based on Fisher-KPP reaction-diffusion PDEs (GliODIL, LOTI, PINN-GBM, LMI), while the second family of models directly predicts regions of subsequent recurrence rather than an underlying tumor cell concentration (GlioMap, U-Net, nnU-Net):

- GliODIL is a glioblastoma growth model that predicts a spatiotemporal distribution for the underlying tumor cell concentration. It combines a data loss term with a reaction-diffusion physics term obtained by discretizing the Fisher-KPP PDE according to the Optimizing a Discrete Loss (ODIL) framework to leverage gradient descent and automatic differentiation with deep-learning optimizers^30,64^. In this way, both data and physics-based constraints are softly assimilated into the solution.
- Lightweight Optimization for Estimating Tumor Infiltration (LOTI) estimates a tumor cell concentration by fitting it to the MRI-derived tumor segmentation while enforcing a smooth solution. It uses the Adam optimizer to minimize a combined physics and data loss term, achieving fast runtimes. While the data loss drives the solution to match the MRI-visible tumor, the Dirichlet Energy loss term penalizes large spatial gradients, encouraging physically plausible, diffusion-like solutions as expected from reaction-diffusion models^32^.
- PINN-GBM uses a physics-informed neural network (PINN)^65^ to solve the inverse Fisher-KPP problem and infer patient-specific parameters and the tumor cell concentration from MRI images. First, patient-specific growth parameters are estimated via grid search, and the Fisher-KPP PDE is solved with these parameters using a finite difference method (FDM) together with the diffuse domain method to handle boundary conditions^66^. The solution is then used to pre-train a fully connected neural network that represents the tumor cell concentration in the output layer. Finally, the network is fine-tuned on the segmentation data with trainable patient-specific parameters in the loss function, yielding the final tumor cell concentration and growth parameters^29^.
- Learn-Morph-Infer (LMI) trains a convolutional neural network on simulated data to learn the inverse mapping from MRI to growth parameters in a PDE, such as diffusion coefficient and proliferation rate. The parameters are then used to generate a tumor cell concentration via a conventional PDE solver. The framework also supports more complex PDEs considering mass effect beyond the typical reaction diffusion equations^26^.
- GlioMap is a voxel-wise recurrence prediction model focused on the peritumoral brain region. It extracts voxel-wise radiomic features from MRI modalities, including ADC, to estimate the recurrence probability for each voxel using ML classifiers^34,35,67–69^. Training was performed using undersampling to address the strong class imbalance between peritumoral and recurrence voxels, and a distance-based correction factor was applied to penalize the predicted probabilities for voxels far from the core with a sharp drop-off at 15 mm into the peritumoral edema.
- nnU-Net is a self-configuring framework that was used as a baseline^70^. The baseline nnU-Net was trained using 3-fold cross-validation on our combined dataset, yielding 162 training and 81 test cases per nnU-Net training fold. The data splits were stratified according to the original datasets to account for domain shift across centers. Inputs were the pre-operative tumor segmentation and tissue maps.

The PREDICT-GBM repository contains containerized implementations and instructions for including new models, enabling straightforward extension. Table 3 summarizes runtime and memory requirements. Although GliODIL has significantly longer runtimes, it is also the only model that directly yields a temporal distribution.

**Table 3:** Inference time per case, total training time, and memory requirements during inference for different models

Model	Inference	Training	Memory
LOTI	~ 1 min	–	~ 2 GB
GliODIL	~ 50 min	–	~ 18 GB
U-Net	< 1 s	~ 50 h	~ 1 GB
PINN-GBM	~ 20 min	–	~ 20 GB
LMI	~ 5 min	~ 130 h	~ 1 GB
nnU-Net	~ 5 s	~ 50 h	~ 2 GB

Inference and training times are reported on a Quadro RTX 8000 GPU.

### Custom U-Net

Our custom U-Net is trained using a deep-ensemble strategy with three ensemble members, each using different random initialization and randomized data shuffling^71,72^. For each ensemble member, we trained five U-Nets on distinct train/validation/test splits, resulting in a total of 15 trained U-Nets. To obtain unbiased out-of-sample predictions for all patients, each prediction is generated from the model for which the corresponding patient belongs to the held-out test set, ensuring that all results are obtained on unseen data. We used an extended dataset of 327 patients, yielding 197 training, 65 validation, and 65 test cases per split, stratified by dataset origin. The extended training dataset is supplemented with 84 additional patients from the publicly available datasets Clinical Proteomic Tumor Analysis Consortium Glioblastoma Multiforme Collection (CPTAC-GBM)^73^, Ivy Glioblastoma Atlas Project (IVYGAP)^74^, The Cancer Genome Atlas Glioblastoma Multiforme (TCGA-GBM)^75^, and Multi-parametric magnetic resonance imaging scans for de novo Glioblastoma patients from the University of Pennsylvania Health System (UPENN-GBM)^76^. The additional datasets (CPTAC-GBM, IVYGAP, TCGA-GBM, and UPENN-GBM) are not included in our public data release due to licensing restrictions. The network uses a base of 32 features with a softmax output and instance normalization^77^ to avoid contrast shifts across batches. We used the following inputs: pre-operative tumor segmentation, white matter, gray matter, and CSF probability maps, pre-operative T1c, FLAIR, and ADC scans. ADC was available for 170 patients, with the remainder zero-filled. The binarized recurrence segmentation, registered to pre-operative space, served as ground truth. Channel dropout was used to minimize the impact of missing inputs, along with a data augmentation framework comprising image intensity augmentations (downsampling, intensity scaling, Gaussian smoothing, Gaussian noise, gamma augmentation, and contrast transformation) and synchronous geometry augmentations (random flipping and affine transformations applied jointly to images, brain mask, and labels)^70^. Random Gaussian edge softening^78^ was used to reduce the impact of label noise, transforming the binary segmentations into continuous probability maps. The U-Net was trained using a modified composite loss function inspired by ref. ^36^, combining a binary cross-entropy (BCE) loss with an individualized progression coverage coefficient (PCC) loss derived from the Tversky loss. The PCC loss dynamically adjusts the loss hyperparameters for each patient based on tumor size to address class imbalance. BCE and PCC were weighted equally (0.5 each); recurring edema voxels were weighted 0.25, and recurring enhancing-core and necrosis voxels 0.75, and the loss was masked to zero outside the brain. A grid search yielded a mini-batch size of 7 and a learning rate of 1 × 10^−4^ (Adam, weight decay 1 × 10^−4^). Predictions were post-processed with Gaussian smoothing (*σ* = 1). Finally, to limit deviation from the guideline-based comparator, the model output was linearly combined with a distance transform of the tumor-core center of mass (weights 0.2 and 0.8, respectively). All hyperparameters and the post-processing design were tuned on the validation split of each fold.

### Radiation plans

We compared model-derived target volumes against a margin-based RT target definition. We used a simplified guideline-based 15-mm expansion as a geometric comparator, referred to as the SOC comparator for brevity. This SOC target volume was generated by isotropically dilating the segmented tumor core by 15 mm within the brain mask, consistent with contemporary guideline-based CTV expansions^8^. We emphasize that the SOC is a simplified geometric surrogate evaluated on pre-operative anatomy and is not intended to reproduce full postoperative radiotherapy planning (dose, organ-at-risk constraints, or cavity geometry). For models producing continuous voxel-wise scores, either tumor cell concentration maps (GliODIL, LOTI, PINN-GBM, LMI) or recurrence probability maps (GlioMap, U-Net, nnU-Net), we constructed iso-volumetric targets by selecting the top-*k* highest-scoring voxels such that the resulting target volume matched the SOC volume. This iso-volumetric constraint prevents trivial performance gains via volume inflation and forces models to redistribute a fixed treatment volume toward higher-risk regions^79^. For GlioMap, which provides scores only in the peritumoral region, we added the tumor core to the GlioMap score map prior to top-*k* selection. When a model output contained fewer non-zero (or positive) voxels than required to reach the SOC volume, we expanded the prediction isotropically by binarizing the output, computing a distance transform, and adding voxels in order of increasing distance to the predicted region until iso-volumetry was achieved.

### Recurrence definitions and evaluation

Evaluation was performed by computing the recurrence coverage in the pre-operative reference space using the longitudinal transforms described above. We report two recurrence definitions: (i) enhancing recurrence, and (ii) enhancing plus necrotic recurrence (recurrence core). For each definition, recurrence coverage, or equivalently, the sensitivity, was computed as:

1 $${\rm{Coverage}}=\frac{| {V}_{target}\cap {V}_{rec}| }{| {V}_{rec}| }=\frac{{\rm{TP}}}{{\rm{TP}}+{\rm{FN}}}={\rm{Sens.}}$$

where *V*_*target*_ is the evaluated target volume (SOC or model-based) and *V*_*rec*_ is the corresponding follow-up segmentation. Foci were defined as connected components of volume ≥0.1 cm^3^ separated from neighboring components by at least 1 cm.

### Post-operative evaluation

To assess validity in the clinically realistic post-operative setting, we re-evaluated all models in post-operative reference space for the subset of patients with an available post-operative exam (*N* = 189). These images were processed through the same pipeline as the pre-operative cohort; the follow-up recurrence segmentation was deformably registered to post-operative space, and the model-derived plans were propagated from pre-operative to post-operative space via the corresponding longitudinal transform.

### Statistical analysis

Because recurrence coverage values are bounded and frequently concentrated near 0 or 1, recurrence coverage cannot be assumed to be normally distributed. Thus, paired Wilcoxon signed-rank tests were applied to compare per-patient recurrence coverage between each model-based target and the SOC target under each recurrence definition. To control the false discovery rate at *α* = 0.05 across the multiplicity introduced by evaluating multiple models against the guideline-based comparator, the Benjamini-Hochberg procedure^80^ was applied to three families such that overlapping cohorts are never pooled into the same family: (i) PREDICT-GBM (m=5), (ii) RHUH, TUM-GBM, and LUMIERE (m=15), and (iii) DIFFUSION (m = 6). Statistical significance is declared at Benjamini-Hochberg adjusted *p* < 0.05.

Paired effect sizes are summarized by the Hodges-Lehmann estimator^81^ with a distribution-free 95% confidence interval obtained by inverting the Wilcoxon signed-rank test^82^. Both the Hodges-Lehmann estimator and the inverted CI exclude zero paired differences from their construction, following Wilcoxon’s original zero-handling convention^83^, which keeps the effect-size estimation consistent with the paired Wilcoxon signed-rank tests used for *p*-values and is appropriate in this iso-volumetric setting where model and SOC plans frequently produce identical (saturated) coverage on individual patients.

## Supplementary information

Supplementary information

## Data Availability

To foster reproducibility, we provide the exact list of patients and the modalities identified for each public dataset at github.com/BrainLesion/PredictGBM. Furthermore, we release skull-stripped MRI exams for 142 subjects from the TUM-GBM dataset, ensuring that all 243 patients are publicly available. By providing this information, anyone with access to the public datasets can reproduce our experiments while protecting patient anonymity. In addition to the MRI exams, we provide the processed tumor segmentations, tissue segmentations, and growth model tumor cell maps as NIfTI files at huggingface.co/datasets/LZimmer/PREDICT-GBM. The LUMIERE dataset is publicly available at doi.org/10.6084/m9.figshare.c.5904905 and the RHUH dataset is publicly available at cancerimagingarchive.net/collection/rhuh-gbm/. The 84 additional training patients used to extend the cohort are drawn from publicly accessible TCIA collections (CPTAC-GBM, IvyGAP, TCGA-GBM, UPENN-GBM); their data-use agreements permit downstream research use but do not permit redistribution of the raw or derived images. We therefore release the exact patient lists and preprocessing configuration required to reconstruct the extended training set from the TCIA originals at huggingface.co/datasets/LZimmer/PREDICT-GBM, together with pretrained U-Net weights for all 15 ensemble members at huggingface.co/LZimmer/PREDICT-GBM-Models. Exact end-to-end retraining of the full U-Net ensemble requires reconstruction of the training set using the provided patient lists for the extended dataset and using our open-source pipeline for pre-processing.

## Code availability

The complete PREDICT-GBM pipeline is publicly available at github.com/BrainLesion/PredictGBM. The repository provides the full end-to-end workflow, including containerized implementations of the evaluated growth models and recurrence prediction models. Detailed documentation and integration guidelines are provided to facilitate the inclusion of new models.
