# Supplementary material for: PREDICT-GBM: A multicenter platform advancing personalized glioblastoma radiotherapy planning
Source: NPJ Digit Med. 2026 Sep 9;9:686. doi: 10.1038/s41746-026-03194-0 (PMC13558737; doi:10.1038/s41746-026-03194-0)
Supplement: Supplementary file 1 [file 41746_2026_3194_MOESM1_ESM.pdf]

Supplementary Information

Supplementary Note 1: Metrics

Figures 1 and 2 present the Dice coefficient and 95 % Hausdorff distance, including median values, for the enhancing recurrence across the combined cohort. PINN-GBM, GliODIL, LOTI, and our custom U-Net achieved slightly higher median Dice scores as well as slightly higher mean Dice scores. Our U-Net achieved the highest mean Dice score (10.67 ± 0.78 compared to SOC 10.34 ± 0.77). For the 95 % Hausdorff distance, SOC achieved the lowest (best) median value of 32.02 mm. The best mean value was achieved by PINN-GBM with 36.44 ± 1.21 mm.

Table 1 reports (a) the median coverage with IQR, (b) the Hodges-Lehmann paired shift with distribution-free 95 % CIs, and (c) the proportion of patients with at least a 5 % absolute coverage improvement over SOC. Per-patient coverage is concentrated near saturation with a heavy tail of low-coverage cases. On PREDICT-GBM the SOC median is 96.39 % against a mean of 77.34 %, so the tail drives mean differences between models. Under the Hodges-Lehmann estimator, the U-Net is the highest positive estimator on every dataset row, with CIs excluding zero on TUM-GBM, LUMIERE, DIFFUSION, and PREDICT-GBM. RHUH is the only cohort where its CI spans zero, consistent with the smaller $N = 40$ and the near-saturated SOC median (94.16 %). GliODIL is second-ranked with CIs excluding zero on TUM-GBM, DIFFUSION, and PREDICT-GBM. LOTI and PINN-GBM cluster around zero with CIs largely spanning it, while LMI and the nnU-Net baseline are strongly negative throughout. Tied paired differences were excluded prior to rank construction, following Wilcoxon's original procedure [83]. Under Pratt's alternative [84], which retains zeros in the ranking, all 95 % CIs in Table 1b would span zero. Table 1b is therefore to be read as reporting paired effect sizes on the subset of patients for whom the model and SOC produce different coverages. We adopt this convention, as mentioned in the Methods section, because zero paired differences predominantly reflect coverage saturation rather than informative agreement (as indicated by Table 1a), and because it matches the paired Wilcoxon signed-rank tests. The proportion of patients with ≥ 5 % improvement over SOC is ~ 15–20 % for the top models, peaking at 21.3 % for the U-Net on LUMIERE.

Supplementary Note 2: Subgroup analysis

To obtain a more fine-grained view of model performance across patient subgroups, we stratified recurrence coverage by primary tumor size, center-of-mass (CoM) distance between primary tumor and recurrence, and the number of foci in both the primary tumor and the recurrence. Results are shown in Figure 4.

Both model-based plans and the SOC achieved lower coverage for small primary tumors. Coverage also decreased markedly for distant recurrences (CoM distance ≥ 30 mm; $N = 46$). In this regime, model-based plans showed a modest advantage over the SOC. This is expected because the SOC is constrained to a uniform margin around the primary tumor, while model-based plans can redistribute volume toward more distant high-risk regions. Coverage further decreased with the number of foci, for both primary tumors and recurrences. Under the above definition, multi-focal primary tumors were rare in our cohort ($N = 10$), whereas multi-focal recurrences were considerably more common ($N = 67$).

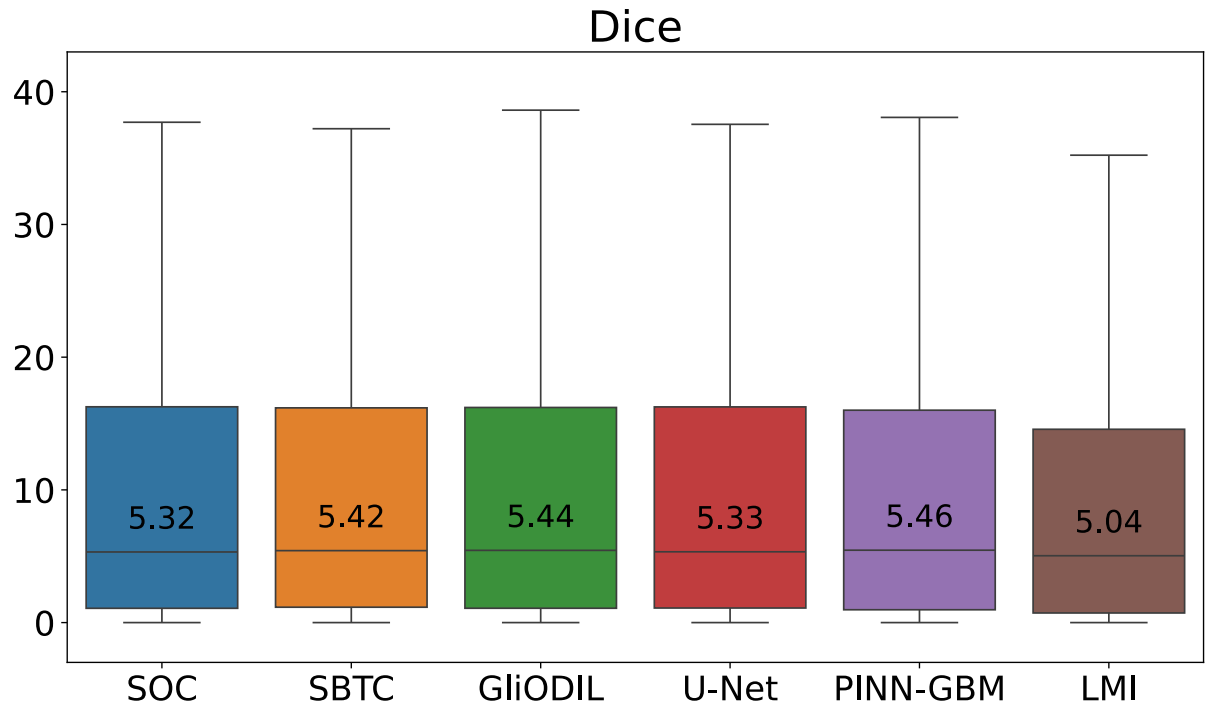

Supplementary Figure 1 Dice coefficient across the combined cohort for the enhancing recurrence.

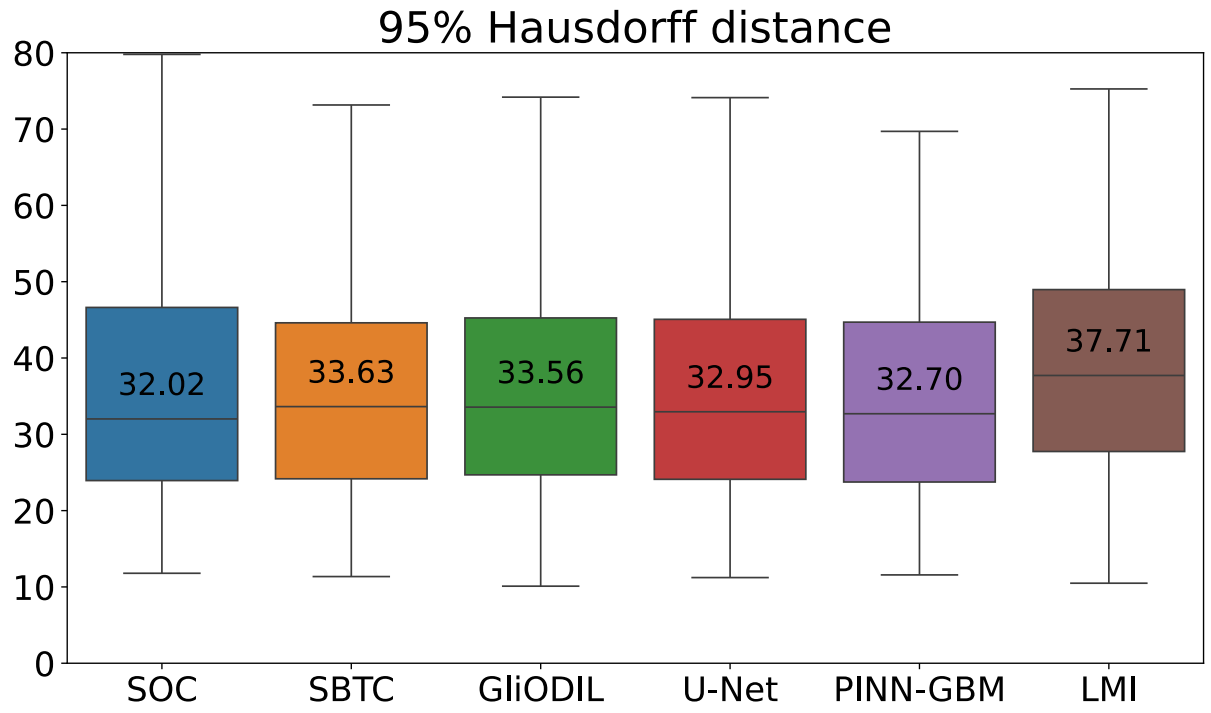

Supplementary Figure 2 95 % Hausdorff distance across the combined cohort for the enhancing recurrence.

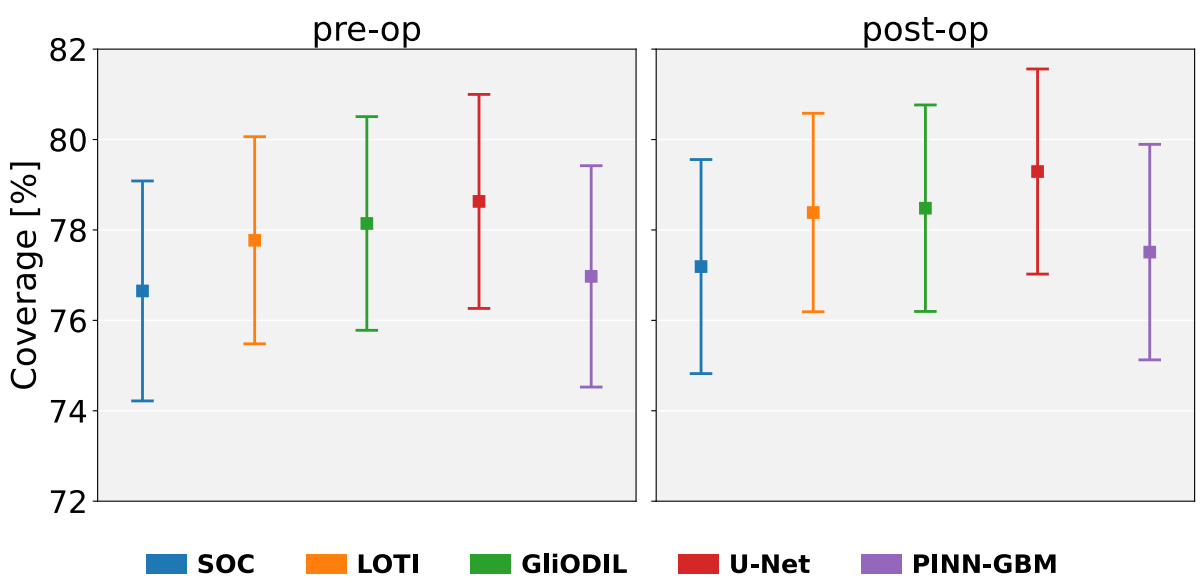

Supplementary Figure 3 Coverage of enhancing recurrence on our combined dataset evaluated in pre-op and post-op space. Error bars denote the standard error of the mean.

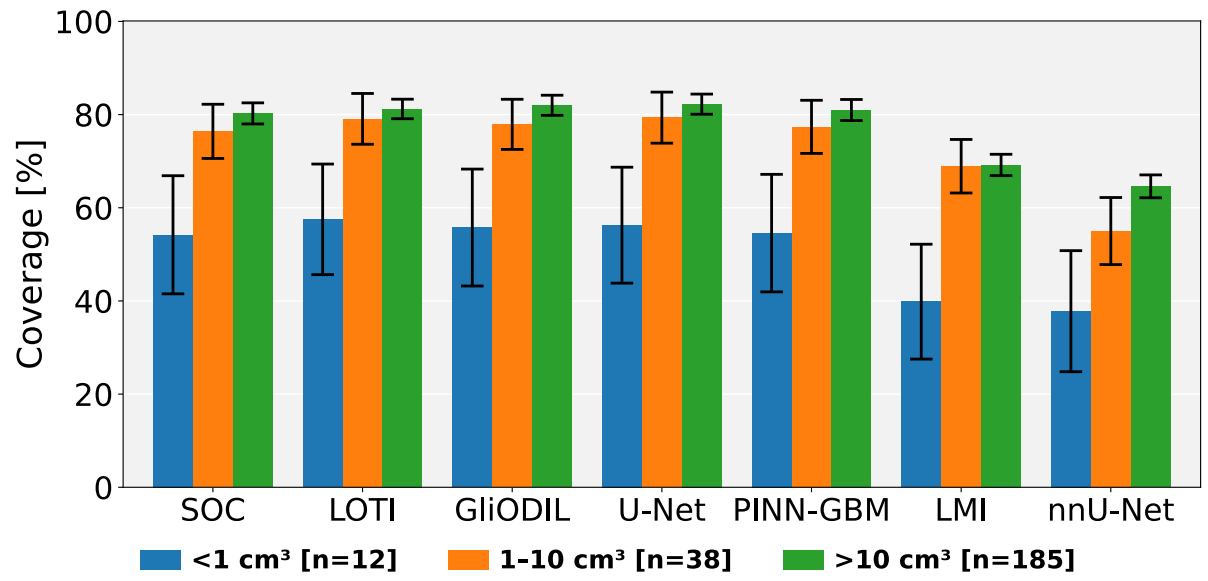

(a) Mean coverage by tumor volume.

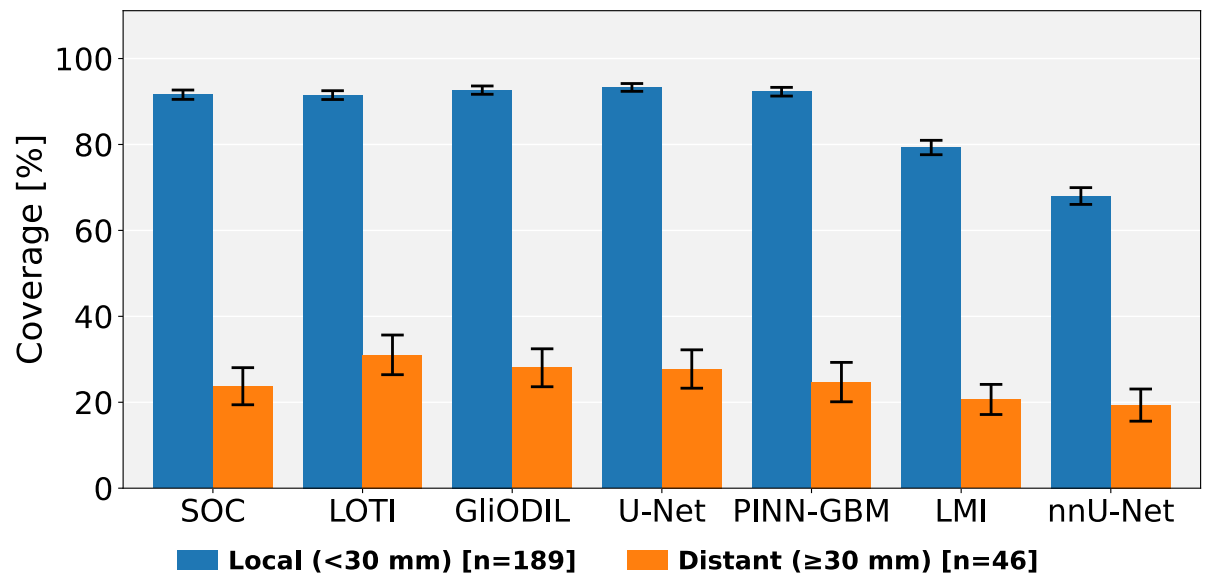

(b) Mean coverage by recurrence distance.

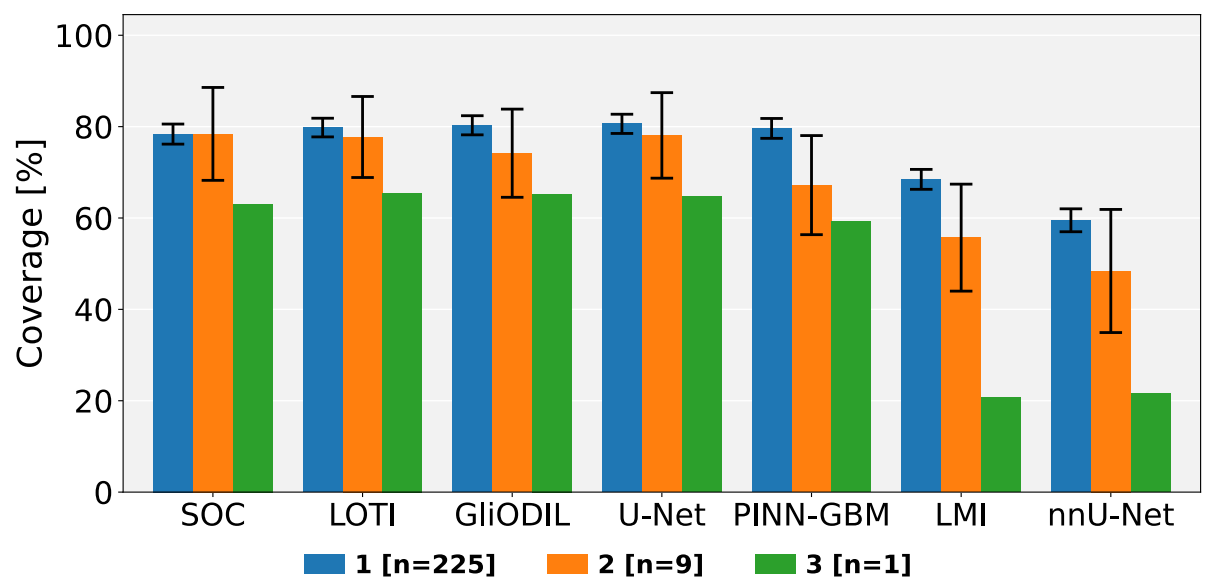

(c) Mean coverage by number of tumor foci.

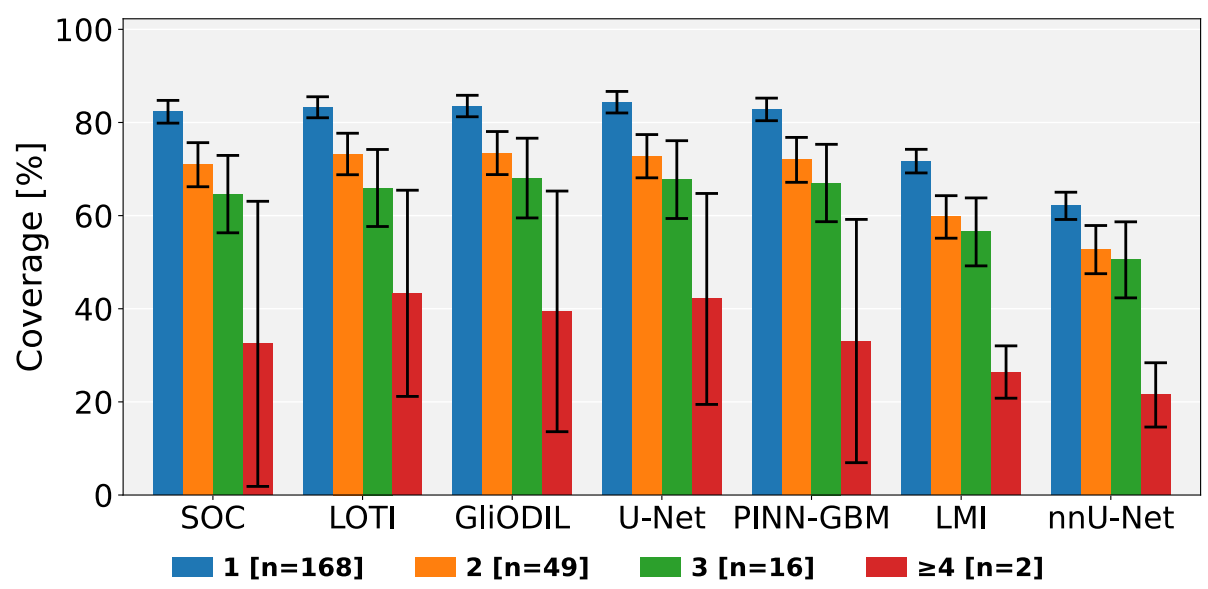

(d) Mean coverage by number of recurrence foci.

Supplementary Figure 4 Mean coverage and standard error for the enhancing+necrotic recurrence by (a) tumor volume at the first MRI exam, (b) center-of-mass distance between primary tumor and recurrence, (c) number of primary tumor foci, (d) number of recurrence foci. Foci were defined as connected volumes $\geq 0.1\,\text{cm}^3$ and minimum distance of 1 cm to neighboring foci.

Dataset	SOC	LOTI	GliODIL	U-Net	PINN-GBM	LMI	nnU-Net	GlioMap
RHUH	94.16 [83.67, 100.00]	91.70 [80.78, 98.89]	94.05 [83.30, 99.68]	**95.27** [**84.32**, **99.90**]	94.75 [81.14, 99.97]	77.47 [58.65, 89.34]	57.71 [41.57, 74.89]	–
TUM-GBM	98.30 [79.88, 100.00]	99.03 [80.07, 100.00]	98.97 [82.77, 100.00]	**99.37** [**83.65**, **100.00**]	98.84 [82.00, 100.00]	86.08 [42.26, 97.21]	69.49 [35.44, 85.94]	–
LUMIERE	84.20 [40.64, 100.00]	85.62 [43.98, 99.98]	87.42 [40.33, 99.93]	**91.20** [**39.76**, **99.98**]	88.89 [40.37, 100.00]	75.89 [39.66, 96.14]	68.18 [34.16, 83.51]	–
DIFFUSION	98.15 [82.83, 100.00]	99.25 [87.21, 100.00]	99.28 [89.53, 100.00]	**99.42** [**87.19**, **100.00**]	98.94 [83.68, 100.00]	86.64 [49.65, 97.34]	76.44 [38.91, 86.77]	97.57 [78.28, 100.00]
PREDICT-GBM	96.39 [69.80, 100.00]	94.65 [72.71, 100.00]	97.32 [71.50, 100.00]	97.25 [74.31, 100.00]	**97.80** [**68.26**, **100.00**]	79.56 [44.28, 96.28]	68.10 [35.99, 82.02]	–

(a) Median coverage and IQR for the enhancing recurrence.

Dataset	LOTI	GliODIL	U-Net	PINN-GBM	LMI	nnU-Net	GlioMap
RHUH	−1.98 [−4.28, 0.23]	0.86 [−0.48, 3.51]	**1.16** [**−0.80**, **3.96**]	1.16 [−0.63, 3.72]	−12.21 [−17.77, −6.91]	−25.15 [−30.47, −15.98]	–
TUM-GBM	1.08 [−0.38, 2.89]	1.82 [0.71, 3.34]	**2.24** [**0.89**, **4.09**]	0.54 [−0.21, 1.85]	−9.42 [−15.15, −5.35]	−14.91 [−19.97, −10.81]	–
LUMIERE	0.18 [−2.07, 3.24]	0.24 [−1.48, 2.81]	**3.12** [**0.67**, **6.00**]	0.42 [−1.64, 2.38]	−4.18 [−7.49, −1.13]	−8.29 [−11.83, −6.04]	–
DIFFUSION	2.17 [0.23, 4.55]	1.99 [0.87, 3.80]	**2.38** [**0.93**, **4.29**]	1.08 [−0.11, 2.39]	−8.63 [−14.37, −4.63]	−14.17 [−19.55, −9.85]	0.28 [−2.07, 2.56]
PREDICT-GBM	0.19 [−0.86, 1.51]	1.27 [0.47, 2.35]	**2.23** [**1.15**, **3.56**]	0.62 [−0.08, 1.60]	−8.29 [−11.48, −5.70]	−14.03 [−18.41, −11.28]	–

(b) Hodges-Lehmann estimator for paired effect sizes with distribution-free 95 % CI (tied paired differences excluded).

Dataset	SOC	LOTI	GliODIL	U-Net	PINN-GBM	LMI	nnU-Net	GlioMap
RHUH	–	10.00	**20.00**	20.00	17.50	10.00	5.00	–
TUM-GBM	–	16.90	16.90	**18.31**	13.38	12.77	8.45	–
LUMIERE	–	14.75	13.11	**21.31**	13.11	14.75	8.20	–
DIFFUSION	–	**20.54**	16.96	19.64	14.29	13.39	8.93	15.18
PREDICT-GBM	–	15.23	16.46	**19.34**	13.99	12.81	7.82	–

(c) Percent of patients with improvement of at least 5 % in coverage over the guideline-based comparator for the enhancing recurrence.

Supplementary Table 1. Coverage-derived metrics for the enhancing recurrence by model and dataset: (a) median coverage and IQR (ties included), (b) Hodges-Lehmann estimator for paired effect sizes with distribution-free 95 % CI, computed over the subset of patients where model and SOC coverages differ (tied paired differences excluded), (c) percent of patients with at least 5 % coverage improvement over the guideline-based comparator.
